# Correlation between peripheral blood α1-MG, DNMT1 expression, and the severity of diabetic nephropathy renal pathological damage

**DOI:** 10.1097/MD.0000000000035409

**Published:** 2023-10-20

**Authors:** Liang Jin, Chao Niu, Yulong Ni

**Affiliations:** a Department of Hand and Foot Surgery, Xingtai People’s Hospital, Xingtai, Hebei, People’s Republic of China.

**Keywords:** 1-microglobulin (α1-MG), diabetic nephropathy, DNA methyltransferase 1 (DNMT1), severity of renal pathological damage, vascular endothelial growth factor (VEGF)

## Abstract

To explore the correlation between peripheral blood α1-microglobulin (α1-MG) and monocyte DNA methyltransferase 1 (DNMT1) expression and the severity of renal pathological damage in diabetic nephropathy (DN). The study group comprised 100 patients with DN who underwent treatment at our hospital from January 2022 to January 2023, while the control group consisted of 50 patients with uncomplicated diabetes. The relative expression levels of peripheral blood α1-MG and DNMT1 were compared between the 2 groups of patients. Additionally, the levels of vascular endothelial growth factor (VEGF) were measured, and the diagnostic value of DN was explored using ROC curves. Furthermore, the correlation between the aforementioned indicators and the severity of renal pathological damage in the patients of the study group was analyzed. Compared to the patients in the control group, the patients in the study group showed increased relative expression levels of peripheral blood α1-MG and DNMT1, as well as elevated levels of VEGF (*P* < .05). The diagnostic value of peripheral blood α1-MG, DNMT1 relative expression levels, and VEGF levels for DN was explored using ROC curves. The AUC values were 0.907, 0.923, and 0.936, respectively (*P* < .05). The relative expression levels of peripheral blood α1-MG, DNMT1, and VEGF levels in DN patients increase with the elevation of the interstitial fibrosis and tubular atrophy scoring (IFTA) score, showing a positive correlation with r-values of 0.651, 0.710, and 0.628, respectively (*P* < .05). The relative expression levels of peripheral blood α1-MG, DNMT1, and VEGF levels in DN patients increase with the elevation of the interstitial inflammation score, showing a positive correlation with r-values of 0.771, 0.633, and 0.678, respectively (*P* < .05). The relative expression levels of peripheral blood α1-MG, DNMT1, and VEGF levels in DN patients increase with the elevation of the glomerular grading, showing a positive correlation with r-values of 0.714, 0.609, and 0.677, respectively (*P* < .05). The expression levels of peripheral blood α1-MG, DNMT1, and VEGF are significantly elevated in patients with DN. These levels show a positive correlation with the IFTA score, interstitial inflammation score, and glomerular grading, contributing to the diagnosis and assessment of DN.

## 1. Introduction

Diabetic nephropathy (DN) is one of the most common and severe complications of diabetes, and it is a major cause of chronic kidney disease.^[1,2]^ Research studies have shown that approximately 465 million adults worldwide are affected by diabetes, with about 40% of patients at risk of developing kidney complications. Furthermore, around 20% to 40% of diabetes patients will progress to DN. DN is responsible for approximately 1 million deaths annually and is considered one of the significant factors contributing to the increased global mortality rate.^[3,4]^ It is of great significance to assess the severity of renal pathological damage in DN patients and implement early and effective intervention measures to improve prognosis and enhance quality of life. α1-microglobulin (α1-MG) is a glycoprotein synthesized by the liver and serves as an important biomarker for inflammation and oxidative stress.^[5]^ Some studies have indicated a close association between α1-MG and the occurrence and progression of DN.^[6]^ DNA methyltransferase 1 (DNMT1) is an important enzyme involved in DNA methylation and plays a crucial role in maintaining DNA methylation patterns and gene expression.^[7]^ Studies have shown that the expression levels of α1-MG and DNMT1 are increased in DN and may be involved in the development of DN.^[8]^ However, there is currently no consensus on the correlation between these 2 factors and the severity of DN pathological damage. To investigate the correlation between peripheral blood α1-MG, DNMT1 expression, and the severity of renal pathological damage in DN, this study compared the relative expression levels of α1-MG and DNMT1 in DN patients with those in patients with uncomplicated diabetes. The study findings are reported as follows.

## 2. Data and methods

### 2.1. General data

The study was approved by the Ethics Committee of Xingtai People Hospital. From January 2022 to January 2023, 100 patients with DN who received treatment at our hospital were included in the study group. Additionally, 50 patients with uncomplicated diabetes were selected as the control group. General information from both groups of patients was collected and compared (*P* > .05) (Table 1).

**Table 1:** Comparison of general information between the 2 groups [$\bar{x}$±s, n/(%)].

Group	Age (yr old)	Gender (n/%)		BMI (kg/m^2^)	Course of disease (d)	Systolic pressure (mm Hg)	Diastolic pressure (mm Hg)
		Male	Female				
Control group (n = 50)	52.78 ± 8.79	26 (52.00)	24 (48.00)	22.13 ± 1.78	7.33 ± 2.05	123.16 ± 11.29	80.11 ± 7.64
Study group (n = 100)	53.22 ± 8.99	57 (57.00)	43 (43.00)	22.45 ± 1.55	6.98 ± 2.61	124.05 ± 12.04	80.56 ± 6.98
χ^2^/*t*	−0.157	0.337		−1.093	0.85	−0.435	−0.351
*P*	.876	.561		.276	.397	.664	.726

Inclusion criteria: The enrolled patients were in line with the WHO diagnostic criteria for diabetes^[9]^: fasting blood glucose levels greater than or equal to 7.0 mmol/L, random blood glucose greater than or equal to 11.1 mmol/L. Patients in the study group met the diagnostic criteria for DN in the “Clinical Guidelines for the Prevention and Treatment of Diabetic Kidney Disease in China,”^[10]^ and urinary albumin excretion rate was ≥20 µg/min. Patients with complete clinical data, volunteered to join the study, and signed informed consent.

Exclusion criteria: Patients with acute or chronic glomerulonephritis, polycystic kidney disease, other primary or secondary kidney disease. There are other systemic diseases, such as heart disease, malignant tumor, etc. Patients with kidney surgery or transplantation. Those who participated in other clinical trials. Patients took immunosuppressive agents or nephrotoxic drugs within 3 months before enrollment.

### 2.2. Methods

All participants were required to provide a fasting blood sample of 5ml from the antecubital vein in the early morning. The samples were then centrifuged at 3000 rpm for 10 minutes, and the upper serum layer was collected for further analysis. The relative expression levels of α1-MG, DNMT1, and the level of vascular endothelial growth factor (VEGF) were measured using enzyme-linked immunosorbent assay kits. All kits were purchased from Roche Diagnostics in Shanghai.

### 2.3. Observed indicators

1. The relative expression levels of peripheral blood α1-MG, DNMT1, and VEGF levels were recorded for both groups of patients. The diagnostic value of these markers for DN was explored using ROC curves.
2. The severity of renal pathological damage in patients was assessed using interstitial fibrosis and tubular atrophy scoring (IFTA),^[11]^ interstitial inflammation scoring,^[12]^ and glomerular grading.^[13]^ The correlation between these assessments and the expression levels of peripheral blood α1-MG, DNMT1, and VEGF levels was explored.
3. The IFTA evaluation criteria are as follows: Patients are assessed on a scale of 0 to 3. Grade 0 indicates no significant interstitial fibrosis and tubular atrophy. Grade 1 represents abnormal renal tissue with a volume of <25%. Grade 2 corresponds to abnormal renal tissue comprising approximately 25% to 50% of the total volume. Grade 3 indicates abnormal renal tissue comprising more than 50% of the total volume.
4. The interstitial inflammation scoring criteria are as follows: Patients are graded based on the degree of inflammatory response. Grade 0 indicates no inflammatory response. Grade 1 represents mild inflammatory response. Grade 2 corresponds to moderate inflammatory response. Grade 3 indicates severe inflammatory response.
5. Glomerular grading is as follows: To evaluate the severity of DN in patients with: no significant lesions in patients with normal glomerular performance for grade I, patients with microalbuminuria for 30 to 300 mg/24 h, glomerular hypertrophy for grade II, albuminuria >300 mg/24 h, glomerular obvious lesions for grade III, patients with severe glomerular lesions, renal dysfunction for grade IV, patients with end-stage renal disease, requiring dialysis or kidney transplantation for grade V.

### 2.4. Statistical processing

SPSS23.0 software was used for data processing. Enumeration data such as gender were expressed as (n/%) for chi-square test. The measurement data such as the relative expression of α1-MG, DNMT1 and VEGF in peripheral blood of patients were expressed as ($\bar{x}\pm s$), and the independent sample *t* test was performed. Person correlation analysis was used to analyze the correlation between IFTA score, interstitial inflammation score, glomerular grading and peripheral blood α1-MG, DNMT1 expression and VEGF level. The test level was α = 0.05.

## 3. Results

### 3.1. Correlation analysis the comparison of relative expression of α1-MG and DNMT1 and the level of VEGF in peripheral blood with Peripheral blood glucose level

Compared to the patients in the control group, the patients in the study group showed increased relative expression levels of peripheral blood α1-MG, DNMT1, and VEGF levels (*P* < .05). The relative expression levels of α1-MG, DNMT1 and VEGF in peripheral blood of DN patients were not correlated with peripheral blood glucose levels. The r-values were 0.134, −0.014, 0.024. See Tables 2 and 3

**Table 2:** The comparison of relative expression of α1-MG and DNMT1 in peripheral blood and the level of VEGF between the 2 groups ($\bar{x}$±s).

Group	α1-MG (mg/L)	DNMT1 relative expression levels	VEGF (ng/L)	FBG (mmol/L)
Control group (n = 50)	32.78 ± 8.46	0.07 ± 0.02	63.44 ± 7.12	8.7 ± 3.2
Study group (n = 100)	51.88 ± 12.05	0.14 ± 0.03	302.26 ± 22.16	9.2 ± 2.3
*t*	−5.795	−15.652	−82.723	1.097
*P*	<.001	<.001	<.001	.276

α1-MG = α1-microglobulin, DNMT1 = DNA methyltransferase 1, VEGF = vascular endothelial growth factor.

**Table 3:** The correlation between FBG and the relative expression of α1-MG, DNMT1, and VEGF in peripheral blood of patients.

Group	α1-MG (mg/L)	DNMT1 relative expression levels	VEGF (ng/L)	FBG (mmol/L)
Control group (n = 50)	32.78 ± 8.46	0.07 ± 0.02	63.44 ± 7.12	8.7 ± 3.2
Study group (n = 100)	51.88 ± 12.05	0.14 ± 0.03	302.26 ± 22.16	9.2 ± 2.3
*t*	−5.795	−15.652	−82.723	1.097
*P*	<.001	<.001	<.001	.276

α1-MG = α1-microglobulin, DNMT1 = DNA methyltransferase 1, VEGF = vascular endothelial growth factor.

### 3.2. The diagnostic value of peripheral blood α1-MG, DNMT1 relative expression and VEGF level in DN

**Figure 1.**
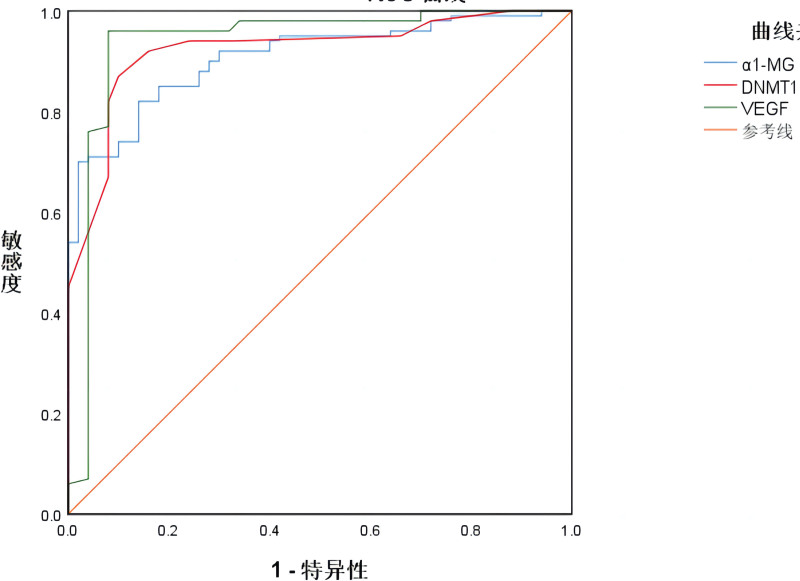
The diagnostic value curve of peripheral blood α1-MG, DNMT1 relative expression and VEGF level in diabetic nephropathy. α1-MG = α1-microglobulin, DNMT1 = DNA methyltransferase 1, VEGF = vascular endothelial growth factor.

ROC curves were used to explore the diagnostic value of peripheral blood α1-MG, DNMT1 relative expression levels, and VEGF levels for DN. The corresponding AUC values were 0.907, 0.923, and 0.936, respectively (*P* < .05). See Table 4 and Figure 1

**Table 4:** The diagnostic value of peripheral blood α1-MG, DNMT1 relative expression and VEGF level in diabetic nephropathy.

Index	AUC	Optimal cutoff value	95%CI		Sensitivity (%)	Specificity (%)	*P*
			Lower limit	Upper limit			
α1-MG	0.907	41.635	0.861	0.953	0.82	0.86	<.001
DNMT1 relative expression levels	0.923	0.105	0.877	0.968	0.87	0.9	<.001
VEGF	0.936	161.495	0.881	0.99	0.96	0.92	<.001

α1-MG = α1-microglobulin, DNMT1 = DNA methyltransferase 1, VEGF = vascular endothelial growth factor.

### 3.3. Correlation analysis of peripheral blood α1-MG, DNMT1 relative expression and VEGF level with IFTA score

In DN patients, the relative expression levels of peripheral blood α1-MG, DNMT1, and VEGF levels increase with the elevation of the IFTA score, showing a positive correlation. The r-values are 0.651, 0.710, and 0.628, respectively (*P* < .05). See Table 5 and Table 6.

**Table 5:** The relative expression levels of α1-MG, DNMT1 and VEGF in peripheral blood of patients with different IFTA scores ($\bar{x}$±s).

IFTA score (score)	Number of cases (n)	α1-MG (mg/L)	DNMT1 relative expression levels	VEGF (ng/L)
0	19	42.78 ± 4.65	0.07 ± 0.02	271.26 ± 21.05
1	26	49.88 ± 7.66	0.09 ± 0.01	299.26 ± 19.26
2	34	55.98 ± 8.13	0.13 ± 0.03	305.26 ± 18.33
3	21	62.39 ± 9.78	0.16 ± 0.05	322.26 ± 19.78

α1-MG = α1-microglobulin, DNMT1 = DNA methyltransferase 1, VEGF = vascular endothelial growth factor.

**Table 6:** The correlation between IFTA score and the relative expression of α1-MG, DNMT1 and VEGF in peripheral blood of patients.

Correlation coefficient	α1-MG	DNMT1 relative expression levels	VEGF
r	0.651	0.71	0.628
*P*	<.001	<.001	<.001

α1-MG = α1-microglobulin, DNMT1 = DNA methyltransferase 1, VEGF = vascular endothelial growth factor.

### 3.4. Correlation analysis of peripheral blood α1-MG, DNMT1 relative expression and VEGF level with interstitial inflammation score

The relative expression levels of peripheral blood α1-MG, DNMT1, and VEGF levels in DN patients increase with the higher interstitial inflammation score, showing a positive correlation. The r-values are 0.771, 0.633, and 0.678, respectively (*P* < .05). See Tables 7 and 8.

**Table 7:** The relative expression levels of α1-MG, DNMT1 and VEGF in peripheral blood of patients with different interstitial inflammation scores ($\bar{x}$±s).

Interstitial inflammation score (score)	Number of cases (n)	α1-MG (mg/L)	DNMT1 relative expression levels	VEGF (ng/L)
0	26	41.88 ± 4.31	0.06 ± 0.03	269.26 ± 21.09
1	39	53.98 ± 5.12	0.12 ± 0.05	298.26 ± 22.06
2	35	59.98 ± 6.78	0.16 ± 0.04	324.26 ± 26.26

α1-MG = α1-microglobulin, DNMT1 = DNA methyltransferase 1, VEGF = vascular endothelial growth factor.

**Table 8:** The correlation between interstitial inflammation score and the relative expression of α1-MG, DNMT1 and VEGF in peripheral blood of patients.

Correlation coefficient	α1-MG	DNMT1 relative expression levels	VEGF
r	0.771	0.633	0.678
*P*	<.001	<.001	<.001

α1-MG = α1-microglobulin, DNMT1 = DNA methyltransferase 1, VEGF = vascular endothelial growth factor.

### 3.5. The correlation between the relative expression of α1-MG and DNMT1 in peripheral blood and the level of VEGF and glomerular grading

The relative expression levels of peripheral blood α1-MG, DNMT1, and VEGF levels in DN patients increase with the higher glomerular grading, showing a positive correlation. The r-values are 0.714, 0.609, and 0.677, respectively. (*P* < .05). See Tables 9 and 10.

**Table 10:** The correlation between glomerular grading and the relative expression of α1-MG, DNMT1, and VEGF in peripheral blood of patients.

Correlation coefficient	α1-MG	DNMT1 relative expression levels	VEGF
r	0.714	0.609	0.677
*P*	<.001	<.001	<.001

α1-MG = α1-microglobulin, DNMT1 = DNA methyltransferase 1, VEGF = vascular endothelial growth factor.

**Table 9:** The relative expression levels of α1-MG, DNMT1 and VEGF in peripheral blood of patients with different glomerular grades ($\bar{x}$±s).

Glomerular grade (grade)	Number of cases (n)	α1-MG (mg/L)	DNMT1 relative expression levels	VEGF (ng/L)
I and II grade	29	42.15 ± 6.77	0.08 ± 0.02	266.29 ± 22.08
III grade	41	52.64 ± 7.45	0.10 ± 0.04	302.41 ± 31.02
IV grade	30	60.88 ± 6.98	0.17 ± 0.06	333.10 ± 29.48

α1-MG = α1-microglobulin, DNMT1 = DNA methyltransferase 1, VEGF = vascular endothelial growth factor.

## 4. Discussion

DN is one of the common chronic complications of diabetes. As the disease progresses, it can lead to glomerulosclerosis and interstitial fibrosis, and in severe cases, it can result in kidney failure.^[14]^ Current research suggests that long-term high blood sugar levels are the major underlying cause of structural and functional abnormalities in the glomeruli and renal tubules, leading to the development of DN. High blood sugar levels can damage the endothelial cells of the glomerular blood vessels, causing expansion and disruption of the intercellular spaces. Additionally, high blood sugar disrupts the charge barrier of the glomerular filtration membrane, leading to increased permeability of the membrane. As a result, large molecules such as proteins can easily pass through the filtration membrane and enter the urine, resulting in proteinuria. Long-term high blood sugar levels can stimulate mesangial cells in the glomeruli to produce an excessive amount of extracellular matrix (ECM). Local deposition of ECM can lead to structural abnormalities in the glomeruli, such as thickening of the glomerular basement membrane, narrowing of the glomerular capillary lumens, and deformation of the glomerular filtration membrane. High blood sugar levels can also lead to the activation of inflammatory responses and increased oxidative stress. This can result in the release of cytokines and infiltration of inflammatory cells, leading to glomerular injury. Additionally, high blood sugar can cause renal artery sclerosis and reduce renal blood flow, leading to inadequate perfusion of the glomeruli. This can result in hypoxia and inadequate nutrient supply to glomerular cells, further exacerbating renal damage.^[15,16]^ Early identification of the pathological damage extent in DN patients is crucial for guiding the selection of clinical treatment strategies, which plays a significant role in improving treatment efficacy and reducing complications. This study aimed to explore the correlation between the relative expression levels of peripheral blood α1-MG, DNMT1 and the extent of renal pathology in DN patients by comparing them with patients with simple diabetes. The findings of this study may provide a theoretical basis for guiding the clinical treatment of DN.

The results of this study showed that the relative expression levels of peripheral blood α1-MG, DNMT1, and VEGF were higher in DN patients compared to patients with simple diabetes. This indicates a correlation between the occurrence of DN and the relative expression levels of peripheral blood α1-MG, DNMT1, and VEGF. In patients with DN, long-term high blood sugar levels lead to inflammation and injury in the glomeruli, resulting in increased release of α1-MG. α1-MG is involved in the pathological process of DN by inhibiting the proliferation of glomerular endothelial cells and promoting cell apoptosis. Therefore, the peripheral blood α1-MG levels are elevated in DN patients. DNMT1 is a key enzyme involved in the DNA methylation process and is responsible for maintaining the stable state of methylated DNA molecules within cells. In DN patients, the hyperglycemic state leads to increased expression and activity of DNMT1, resulting in changes in DNA methylation patterns. These changes include abnormal methylation of genes related to glomerular structure and function, which disrupts gene expression in glomerular cells and affects their function and apoptosis. VEGF, a pro-angiogenic factor, is involved in the process of blood vessel formation. Prolonged hyperglycemia can lead to hypoxia and ischemia in the glomeruli, which stimulates the production and release of VEGF. This, in turn, promotes the proliferation of glomerular endothelial cells and increases vascular permeability as a compensatory response to glomerular hypoxia and ischemia. However, excessive endothelial proliferation and increased permeability can also contribute to the development of DN. The ROC curve analysis in this study revealed that the relative expression levels of α1-MG, DNMT1, and VEGF have diagnostic value for DN, with VEGF showing the highest diagnostic value (AUC 0.936, sensitivity 96.0%, specificity 92.0%). These findings are consistent with the results of a study conducted by Chen et al^[17]^

The elevation of α1-MG can increase the release of inflammatory factors. α1-MG can bind to specific receptors on the surface of immune cells, leading to increased release of inflammatory cytokines such as tumor necrosis factor-alpha (TNF-α), interleukin-1 beta (IL-1β), and interleukin-6 (IL-6) through pathways such as mitogen-activated protein kinase (MAPK) and nuclear factor-kappa B (NF-κB).^[18]^ Elevated levels of these inflammatory factors can further exacerbate glomerular and interstitial damage in the kidneys. DNMT1 also plays an important role in the pathological damage process of the kidneys in patients with DN. In a high glucose environment, intracellular accumulation of advanced glycation end-products and excessive glucose metabolites occurs. These abnormal metabolic products can promote the expression and activation of DNMT1 through the activation of signaling pathways such as RAS and NF-κB.^[19]^ Overexpression of DNMT1 induces methylation of transcription factors and ECM-related genes to regulate ECM synthesis, leading to increased deposition of ECM in glomerular and tubular epithelial cells. The deposition of ECM causes glomerulosclerosis and interstitial fibrosis, further exacerbating renal pathological damage. Additionally, the upregulation of DNMT1 can decrease cellular tolerance to oxidative stress by methylating and inhibiting the expression of antioxidant genes, thereby aggravating kidney damage. In patients with DN, renal microcirculation impairment and tissue ischemia and hypoxia lead to an elevation in VEGF levels through a negative feedback mechanism. Excessive expression of VEGF causes vasodilation in the kidneys, increasing the permeability of both intra- and extra-glomerular blood vessels. This leads to damage to the glomerular filtration membrane and results in proteinuria. The elevated VEGF levels in DN patients dilate the blood vessels in the glomerular endothelial cells and renal tubular epithelial cells, thereby increasing the permeability of the capillaries and allowing plasma proteins to leak into the urine. In addition, elevated levels of VEGF promote angiogenesis, but abnormal neovascularization may not possess the structure and function of normal blood vessels, leading to uneven distribution of blood flow and exacerbating renal tissue ischemia and hypoxia.

This study results demonstrate that in patients with DN, the relative expression levels of α1-MG, DNMT1 in peripheral blood, and VEGF levels are positively correlated with IFTA score, interstitial inflammation score, and glomerular grade. This further confirms the previous observations. IFTA score, interstitial inflammation score, and glomerular grade are commonly used scoring systems to assess the severity of renal pathological damage. The IFTA score is used to assess the degree of interstitial fibrosis and tubular atrophy in the kidneys. A higher score indicates a more severe condition of interstitial fibrosis and tubular atrophy. The interstitial inflammation score is used to evaluate the extent of inflammation in the renal interstitium. A higher score indicates a more pronounced level of interstitial inflammation. The glomerular grade is used to assess the extent of damage to the glomeruli in the kidneys. A higher score indicates more severe glomerular injury. The results of this study indicate a positive correlation between the relative expression levels of α1-MG, DNMT1, and VEGF levels with the severity of renal pathological damage in patients with DN. This finding is consistent with the research conducted by Xu L. et al.^[20]^

In summary, the peripheral blood levels of α1-MG, DNMT1 expression, and VEGF are significantly elevated in patients with DN, and they show a positive correlation with IFTA score, interstitial inflammation score, and glomerular grade. These findings contribute to the diagnosis and assessment of DN.

## Author contributions

**Conceptualization:** Liang Jin.

**Data curation:** Liang Jin.

**Formal analysis:** Chao Niu.

**Investigation:** Chao Niu.

**Methodology:** Chao Niu.

**Supervision:** Yulong Ni.

**Writing – original draft:** Liang Jin.

**Writing – review & editing:** Liang Jin.
